# On the Psychological Basis of the Language of Orators, Poets, and Philosophers

**Published:** 1858-10-01

**Authors:** A. F. Mayo

**Affiliations:** ESQ., BARRISTER-AT-LAW


### Art. III.—ON THE PSYCHOLOGICAL BASIS OF THE LANGUAGE OF ORATORS, POETS, AND PHILOSOPHERS.

BY A. F. MAYO, ESQ., BARRISTER-AT-LAW.

It is my object in this paper to suggest some distinction as to the varying necessities under which the orator, the poet, and the philosopher lie in regard to the possession of an abundant vocabulary. Some light may thence be thrown upon the necessities of the ordinary intercourse of mankind; for no man can talk with his neighbour without evincing the presence of mental ingredients, which in their highest energy become poetry, oratory, or philosophy.

The orator is frequently in want of language under the stringency of that very emotion which he must needs yield to if he is to animate his audience with kindred fire. The danger to which he is exposed is that, if he may not possess such a sufficient repertory of language out of which he may select the words which may paint the passing shade of intensified thought—what happens?—his inspiration may be suddenly chilled in consequence of his intellect being turned into a channel divergent from the main stream of his discourse. He has to let down his plummet into the depths of the ocean of language and laboriously to examine its contents. No longer like a ship careering over the surface, the orator is like the diver, subjected to the heaviest pressure of the element which he fain would master. How often those sympathies are chilled which, if they were rightly seconded by language, would carry the orator to the highest summit of success; unable at the lucky instant to marry his thought to burning words, what happens to him? He falls into some philosophic generality, from the cold trammels of which he is himself unable to escape; and all this from his impotence to find the right word at the right moment.

The consequence is that men of high philosophic power require, if they are to become orators, a copious vocabulary and a ready memory to evoke it, though the want of both is sometimes compensated for in the persuasiveness of an electric sympathy irradiating the most distant portions of the argument. Let me explain this more fully.

An orator who makes use of a general term because he is unable to find a more specific one, becomes himself entangled in that sense of the term which has least to do with the subject in hand, very much as the owner of a vast estate frequently envies the petty freeholder who owns the outlying angle which is wedged into a corner of it. In the same way, a general term ever invites a philosophic mind to explore the remotest boundaries of its meaning. But this meaning is often the very one which has least to do with the subject in hand; and which is therefore also the feeblest in exciting the sympathies either of the orator or his audience. And thus it happens that the electric bond of sympathy which must have otherwise been indefinitely intensified becomes suddenly dissolved. The steed no longer feels the magic touch of a confident and easy sway; that sway has now become vacillating, uncertain, infirm.

Thus the orator has lost the command over his audience regarded as men of like passions with himself; and he can only now address their intellect, for this is the only principle which remains at work within himself. Nature ever throws her strength to the support of the weakest part; and for the moment, the weakest part of the speaker is his vocabulary. During the remainder of his speech, the speaker may still be the dialectician, and even the rhetorician; but he can become an orator again only by igniting his emotiveness from fuel derived from the central storehouse of inflammable associations. And as the captain of a vessel can by the process of tacking avail himself of the aid of nearly every wind that blows; so also the practised speaker can often from sources deep-laid within his own breast, yet most invisible to his audience, replenish the flame of his oppressed oratory.

Yet in this way, to regain a mastery over his audience, the orator must have a mastery over himself. If he cannot suborn it to his purpose of guiding the minds of his audience, the speaker must learn how decently to stifle the philosophic abstraction which perplexes him, he must quell the ambition that seeks in the forum the conquests of the study. But to do this well requires great skill, as much, in fact, as that which is required of a mason to conceal the junctures of his slab. A quick transition unexplained by point or contrast is as injurious to the orator as an obscure or indeterminate idea.

An orator, then, is bound to be acquainted with and thus also to predict the dip, direction, and the sensitiveness of his own intellectual compass; and he must be acquainted with the causes which are likely to deflect it. Thus he will learn in what way, when involved with an unmanageable idea, he may be able to expel it, if not indeed by main force, and the entrance by which it came, at any rate by some other; an orator's mind should in truth be like a theatre, facile in routes of departure as well as of access. Thus, if any idea cannot be suitably entertained, it should have an opportunity for departure, and this without decomposing the others.

A great speech may be likened to a game of chess; the opening of the game affords us the choice of many well-trod paths the immediate consequences of each of which it is not difficult to predict; it is towards the close that the energetic individuality of a great player is called forth.

So, in the commencement of a mountain ascent, the road is fair and open. The routes, whether convergent or divergent, have been scanned beforehand. The foundations of the mountain are comparatively of easy conquest. So, too, to a practical orator, the exordium is a work of art rather than inspiration. It is in the sudden emergencies provoked by the unseen depths of the emotions and thoughts of his audience that his proper prowess is evoked. As the regions of snow and ice are approached, no track will save the traveller from the necessity of labour; though even then superior knowledge will enable him easily to cross ravines where former travellers have perished. And so it is at the climax of the speech, as in scaling the battlements about the mountain-top, that man's energies are strung up to the highest, or, failing in that, are relaxed into despair.

The same general considerations also apply to a poet, but with a difference.

The danger of the orator is, as I have shown, of breaking down, if the harmony between his emotive and logical powers is disturbed. The search for language induces an anxious interrogation of thought which mars the effect of his emotions. The discursive power of the orator varies with the ease and mastery with which he can control his ideas; and I assume that it is in this discursive power alone that the orator has any confidence of being able duly to sustain his strain. On the other hand, the rapidity and harmony of the inventions of the poet depend mainly on the brightness of his associations, which are themselves connected with emotive agencies.

Again, while the loftiest flights of oratory are winged by the emotions, the greatest genius in poetry which the world has seen (I mean of course Shakspeare), sought its most fitting throne in the harmony of Nature herself; and this is ever the appanage of the intellect proper in its function of the imagination.

The associations are ever-present aids to the poet; and far from involving toil in exciting them, require that natural condition of the mind in which the will is passive. Now, the poet seeking no end but inspiration itself, can follow the association where the orator dare not do so, unless he can make them intellectually serve his end; and the consequence of a failure in this I have shown.

There is another reason why an even tenor is more easily attained by the poet than the orator; it is this—that the mental conditions required of the poet lie more in one plane than those required of the orator. For instance, in a poem the tone is either intellectual or emotive, and no sudden emergencies necessitate the rapid changes which are obligatory upon the orator.

Add to this, that the poet can take his own time and recast his piece at leisure, opportunities which are denied to the orator under the stringency of sudden occasion. Thus the intellectual action of the poet, however intense it may be, requires less versatility in its management than is the case with the orator. The ideas of the poet, possessing a more natural flow of their own, the flow of the language corresponds in unity of tone and character; and the mind, in seeking a right expression, is not trammelled by that constant attention to the ideas themselves which the variety of their gesture renders incumbent on the orator.

As a set-off, however, to the poet's freedom from sudden occasions demanding a large and flexible vocabulary, there is in the superior intellectual possession which the ideas of the poet assert over his mind, a compensating necessity for a wealth of language. Sometimes, too, the absence of any immediate necessity to clothe those ideas which are silently matured in retirement, in the garb of language, may surprise the poet with an inability to convert his ideas into that current coin whereby alone they can affect the world; and this, too, at the very moment when they may vanish altogether unless so converted. And thus the poet may be in the plight of a miser who has been so intent upon the idea of his treasure itself, that he has mislaid the key of the very cabinet which contains it.

There are some peculiar considerations affecting philosophy, metaphysics, and deep speculation in general, in regard to their demands for richness in the vocabulary of language.

Let us see how the metaphysician stands in this respect as compared with the orator and the poet taken together.

A poet and an orator who are, both of them, intellectually deficient—the one feeble in his imaginative—the other feeble in his dialectic powers, can each of them succeed better with a given vocabulary than a strong poet or a strong orator, at least in the first instance. For long use and training of their respective endowments will at last give the strong poet and the orator knowledge of their own strength, and they also will obtain the power of making their intellectual energy tell in the production of clearness as much as of profundity of conception, thus enabling them respectively to convert what was at first only a command over processes of thought, into a command over the resources of language; just as the increasing skill of the hardy workman, who became such from mere strength of arm, will at last engage him in delicate manipulations, to which in the first instance he may have been incompetent from deficiency in a sense of touch.

At first, however, orators and poets of genius are apt to evince rather extravagance or faintness, under stress of poverty, as regards the resources of language; and thus with a given stock of the latter, the halt and the lame often succeed where the otherwise vigorous and athletic fail. The man who is not overwhelmed by the immensity of his ideas or his emotions can afford to wait for the proper term with which to describe them. He need not fear that, while he is seeking how to attire them in language, others may possess and usurp their hold upon his attention.

On the contrary, ideas and emotions which are grand or multitudinous, where they cannot find a vent for escape through the portals of language, are too apt to trip up their owners altogether under that confusion of thought which is the legitimate offspring of confusion of terms.

That currency and abundance in the coin of language so necessary for the evolution of poetry and oratory is not required of the metaphysician, or of all those in whose writings, be they historians, biographers, or what not, abstract and philosophic views of life prevail over that which is concrete and ornate.

The metaphysician has, in proportion to his strength, a happy opportunity of waiting for the full development of his ideas before he clothes them in language; for, regarding but few objects comparatively at the moment, he can safely arrest the vehicle of thought. Without this power of arrestation language could not be held in suspense; as it is, he coolly waits till he has found the right word for his purpose.

The ideas of the poet and the orator are often as quick and transient as the "Gay motes which people the sunbeam—as rich bedecked as the woof of Iris." But the results of the philosopher, slow indeed in their incubation, are not evanescent, and, like the cub of an elephant, they are long-lived also.

The case of the poet and the orator may be likened to that of an army which, if it is to move quickly, must have large attendant resources of baggage, animals, and vehicles. Unless, either naturally or artificially, the poet and the orator possess a large fund of language, they are likely to be starved and paralysed by the way.

Thus both the poet and orator, with a view of insuring that rapid transit of the associations which are usually required to stir up their own emotions, are obliged to throw away correctness of phraseology as an encumbrance, or, retaining the latter advantage, to make a sacrifice of the former principle.

Such sacrifices are much more rarely required of the philosopher. The comparative paucity of the ideas excited at any one time by his studies, and their separate clearness and vividity, are such as to insure their depth and permanence; and these qualities confer upon him an immunity from anxiety lest his ideas should glide away from the disc of thought before they have been photographed in language.

For myself I believe that no clear and definite idea need ever be lost to the world for the want of a due vehicle of expression; provided only that, whenever his resources of terminology fail the thinker, he will deign to express his meaning by a definition.

The essence of the idea conveyed by this definition the speaker may gather up into a new term if required. Thus, a man who has lost his hat may throw his cloak over his head. This cloak (answering to the definition) may suggest some covering more suited to the climate than even his hat. New terms are thus the expression of new wants, or sometimes of old wants, for which the acquired resources of language are to the particular thinker unavailable.

It would appear, therefore, that the language required by philosophy consists of terms of art, and definitive expressions, rather than of the common words which are so valuable in illustration. These latter words form a capital without some power of drawing upon which no poet or orator can start.

In the case of the philosopher there are certain words which are the keystone of the solid bridge with which he connects distant provinces of thought. The airy structures of oratory and poetry require, indeed, more various and flexible materials. But none are of such weight, or require such accurary of line, measurement, and equipoise as those which, few in number, are yet indispensable to the philosopher.

Now it is clear that the emotive region in which, more or less, all poets and orators move must be sustained at a certain degree of tension; that is, if their own sympathies and, by contagion, those of their audience are to be duly excited.

In the degree, then, in which this tension is continuous and can be reckoned on, so long does it form a sort of atmosphere exercising a constant pressure upon their intellectual energies; and thus, whatever terms they use, their ideas, by the pressure of emotive associations, are constantly moulded into a proper shape and cohesion; and in this way they harmoniously blend.

In philosophy, however, there is no extrinsic force thus binding up the parts of a whole. Here the cohesion of the ideas must depend on their proper affinities; and these affinities will not tell unless language, which is the exterior covering of the ideas, act as a conductor to such affinities; just as in chemistry bodies possessing the strongest tendency to combination may be kept at a distance by a non-conducting medium.

On these general principles it is that, while wealth of language is peculiarly the food of the poet and the orator, appropriateness of language is the very life-blood of the philosopher, and each term in a definition which he uses is a counter, which ought to denote a given quantity and quality of meaning.

As regards the capital of language with which he is required to commence, the philosopher clearly has this advantage over the poet and the orator,—that, whereas the two latter require the abundant use of concrete terms, to separate which into their elements would deprive their conceptions of all their life, force, relief and symmetry, the philosopher can dispense with them. For the demands of the latter are satisfied, if he can instil into the reader a clear comprehension of his meaning by a definition itself consisting of a multitude of words; and this is a sort of set-off against his need of a large store of words of art.

On the contrary, were an artist to endeavour to resolve his compound ideas into their elements, the central fire of inspiration which united them would become evanescent,—and that which he sought to adorn would seem a multitude of dead atoms and not a glorious compound bound together by a tie which should not be unloosed,—with the philosopher things are different. However deficient in elegance a definition may be, the philosopher, being careless of the emotive sympathies if he can secure the intellect of his reader, can achieve his end by clearness of language alone.

This, then, is my conclusion, that, subject to the necessity of using in philosophy correct terms of art, a man of high capacity may often achieve large results by the application of but a small vocabulary; that is, if he possesses a delicate discrimination of shades of meaning, and a thorough determination, in the use of terms, not to allow the meaning of one term to overlap the other. And though in this respect he may be in some degree at the mercy of the delinquents of former ages, who have stamped such errors with currency, he may neutralize their ill results with proper provisoes and exceptions, and where necessary by new terms.

It must, however, be remembered, that the fusion into the popular mind, and the verification thereby of the speculation of great thinkers, demand a copious vocabulary. For upon the uneducated terms of art fall with an oppressive weight; at the same time their own experiences are denoted by language which pure abstract thinkers regard as familiar and vulgar; and thus great thinkers often enter the market to purchase popularity with their thousand-pound bank-notes, while the common traffickers know no currency except in pence and silver. Hence, then, to be popular, the greatest thinkers must ever take account of the lowest currency by which the thoughts of men are represented; otherwise they run the risk of starving in the midst of fancied wealth, for the want of a medium of exchange.

Yet to a weak mind, indeed, dealing with a vast subject this wealth of words may prove a source of confusion. Just as one of the Argonauts would find himself more at home in navigating a Chinese junk than a full-rigged man-of-war, or the " Great Eastern." Yet a man of inferior calibre, when confining himself more prudently to the examination of single questions, may derive an insight into his subject by a knowledge of all the terms with which it has been canvassed. An ancient Argonaut would navigate a pinnace more easily than the " Great Eastern." But if patient of instruction, he would learn more from the structure of the " Great Eastern" than from that of the pinnace.

Dr. Roget, in the masterly introduction to the invaluable work, " Thesaurus of English Words and Phrases," has shown us how great is the suggestiveness of a large vocabulary by the mere juxtaposition of words of various shades and various meanings, and even how a mere catalogue of names may often suggest relations and interdependencies between ideas; and often to that central idea round which they latently revolve.

Wealth of language, then, like all other wealth rightly wielded, rightly distributed can never do harm; only let each man take a gauge of his own capacity. In so doing he will not attempt to grasp at a larger mass of ideas (of which words are the counters) than his faculties warrant. So will he be able to carry his burden with ease, without confusion, and without losing integral portions of it.

To minds such as Plato's, Bacon's, or Shakspeare's, language must ever halt behind the majestic tread of their genius. Such indeed was the affluence of Shakspeare's thoughts, that, in order to give a vent to them, he had to ransack the English language to its extremest verge, rejoicing in those sharply cut vernacular terms and idioms upon which the stamp of the great Anglo-Saxon intellect is so clearly impressed; in expressions which, though the prejudices of such men as even Johnson and Voltaire have branded them as vulgar, are alive with the fire of nature, and which under the hand of such a master are landmarks by which we may estimate the almost boundless extent of the incomparable experience of the human mind. Just as the force of the Atlantic is more conspicuous as its waves rush into the deep recesses of the shore-hid inlets on the Cornish coast, than when the expanse of the ocean itself is gazed upon. With Shakspeare language is both the sheath in which the sword of thought is hid and the hilt with which it is grasped. Both hilt and scabbard are mean compared with the weapon which they subserve.

For no capital, no stock of that current coin of language which more than serves the wants of common men could suffice the needs of Shakspeare's traffic with the commodities of thought. A mind so vast had to institute a system of credits and exchanges of less tangible material to supply its daring purposes. Such was the pressure of his thoughts upon his language, that they seem to exercise upon it a sort of domination testified by the homage paid by mankind to the new force and virtues impressed on ancient idioms and words.

So vigorous was this silent compression of language to his will, that mankind gives an intuitive assent to his innovations. Here is the triumph of thought over language, of the soul over its embodiment; here is the outbreak of nature overcoming convention. Language, in fine, may stint the greatest minds by its deficiency, yet it cannot oppress them by its redundancy; while to those of humbler lot, rightly used, it is the fruitful mother of its offspring—Thought.

---

